# Evaluation of creatine content and stability from over-the-counter dietary supplement by ion pair chromatography with UV detection

**DOI:** 10.1186/1550-2783-8-S1-P25

**Published:** 2011-11-07

**Authors:** Peter H Tang

**Affiliations:** 1 Departments of Pediatric, Pathology and Laboratory Medicine, Cincinnati Children's Hospital Medical Center and College of Medicine, University of Cincinnati, Cincinnati, OH 45229, USA

## Background

Creatine is an endogenous guanidine compound found in the skeletal muscles and plays an important role in the metabolism of proteins. A perusal of the information available on the Internet concerning creatine revealed that its activity receives a great deal of attention, with much speculation about its ability to increase lean body mass, high-intensity power output, and strength in humans. Many of the entries available on the World Wide Web come from vendors of creatine. However, creatine differs from many other dietary supplements because its use is advocated by many physicians for many indications. Clinical laboratory monitoring of creatine therapy is currently available and uses HPLC-UV. The plasma creatine concentration increases following oral administration of creatine supplement, and the degree of increase is related positively to the dosage. A method has been developed for the determination of creatine in dietary supplements by using ion pair chromatography (IPC) with UV detection. The objectives of this study were (1) to determine the content of creatine in over-the-counter (OTC) dietary supplements, and (2) to evaluate the stability of creatine in aqueous solutions during storage.

## Methods

Samples were dissolved in type II water to obtain an initial creatine concentration of 10 mg/mL. The final creatine concentration in solutions was 1 mg/mL. Two such solutions were kept at room temperature and 2 others at refrigerated condition. Samples were collected on day zero, day 1, day 2, day 7, day 14, day 21, and day 28. Creatine concentration in the diluted sample was determined by IPC. The internal standard used was guanidinoacetic acid (GAA). 20µL of sample was injected onto the IPC. Separations of creatine, GAA and creatinine were achieved by using a 5-µm reversed-phase C18 column (250 x 4.6 mm) and a mobile phase consisting of phosphate buffer (pH = 2.8, 0.045 M), methanol, sodium dodecyl sulfate, and acetonitrile. The flow rate of IPC run was at 1 mL/min and column temperature at 35°C. Peaks of creatine, GAA and creatinine were monitored at 198 nm.

## Results

The method achieved a linear concentration range of 0.01-2 mg/mL. The limit of detection was 0.003 mg/mL. Both within-run and between-run precision for three controls (0.4, 0.8, and 1.6 mg/mL) were lower than 5%. Analytical recoveries were greater than 95%. Some of the OTC products tested contained lower contents of creatine in contrast to their label claims. Greater degradation occurred in room temperature samples as compared with the refrigerated ones. Sixty percent degradation was observed within 28 days for room temperature samples in citric acid solution. However, at refrigerated condition this degradation was around 40%.

## Conclusion

This method is simple and easy to perform with excellent reproducibility, requires only first step of methanol dilution, centrifugation and final water dilution prior to chromatography.

